# Acute effects of wearing compression knee-length socks on ankle joint position sense in community-dwelling older adults

**DOI:** 10.1371/journal.pone.0245979

**Published:** 2021-02-08

**Authors:** Mei Teng Woo, Keith Davids, Jia Yi Chow, Timo Jaakkola

**Affiliations:** 1 Faculty of Sport and Health Sciences, University of Jyväskylä, Jyväskylä, Finland; 2 School of Sports, Health and Leisure, Republic Polytechnic, Singapore, Singapore; 3 Centre for Sports Engineering Research, Sheffield Hallam University, Sheffield, United Kingdom; 4 Physical Education and Sports Science, National Institute of Education, Nanyang Technological University, Singapore, Singapore; Baylor College of Medicine, UNITED STATES

## Abstract

Functional proprioceptive information is required to allow an individual to interact with the environment effectively for everyday activities such as locomotion and object manipulation. Specifically, research suggests that application of compression garments could improve proprioceptive regulation of action by enhancing sensorimotor system noise in individuals of different ages and capacities. However, limited research has been conducted with samples of elderly people thus far. This study aimed to examine acute effects of wearing knee-length socks (KLS) of various compression levels on ankle joint position sense in community-dwelling, older adults. A total of 26 participants (12 male and 14 female), aged between 65 and 84 years, were randomly recruited from local senior activity centres in Singapore. A repeated-measures design was used to determine effects on joint position awareness of three different treatments–wearing clinical compression socks (20–30 mmHg); wearing non-clinical compression socks (< 20 mmHg); wearing normal socks, and one control condition (barefoot). Participants were required to use the dominant foot to indicate 8 levels of steepness (2.5°, 5°, 7.5°, 10°, 12.5°, 15°, 17.5°, and 20°), while standing on a modified slope box, in a plantar flexion position. Findings showed that wearing clinical compression KLS significantly reduced the mean absolute errors compared to the barefoot condition. However, there were no significant differences observed between other KLS and barefoot conditions. Among the KLS of various compression levels, results suggested that only wearing clinical compression KLS (20–30 mmHg) improved the precision of estimation of ankle joint plantar flexion movement, by reducing absolute performance errors in elderly people. It is concluded that wearing clinical compression KLS could potentially provide an affordable strategy to ameliorate negative effects of ageing on the proprioception system to enhance balance and postural control in community-dwelling individuals.

## Introduction

Proprioception, the sense of positioning and movement of the limbs and body in space, plays a crucial role in regulation of locomotion, balance and postural control, through heightening awareness of limb placement in space [1–3]. Functional proprioception is an important aspect of effective human interactions with the environments in which they dwell [4]. Specifically, the soles of the feet and the ankle joints provide important information about the disposition and movement of the body in space, aiding dynamic balance and postural regulation, nested with other tasks and activities such as reaching for objects and negotiating gaps and surfaces in cluttered environments [5]. For example, precise ankle proprioception is required while walking for ensuring safe clearance when stepping over an obstacle to avoid tripping [6].

A decline in static position sense and an increase in movement detection thresholds at the ankle has been reported in the elderly people [7, 8]. Goble et al. (2009) found that older adults made greater ankle positioning errors on targets for located farther away from the ankle’s original position [9]. It was also suggested that elderly fallers had a lower level of proprioceptive perceptual attunement than non-fallers in a lower-limb matching task, which could be a potential indicator for falls [10]. The mechanism of the proprioception acuity degradation is primarily due to the decline in the sensitivity of peripheral mechanoreceptors in the muscles, skin, and joints [9, 11]. Furthermore, it has been reported that a higher incidence of falls due to trips, stumbles and slips were found to be associated with a decline in functional proprioception in the elderly population [4, 12, 13]. To date, researchers have investigated how proprioception of the placement and positioning of the lower extremities could be enhanced by using various lower-limb stimulation intervention strategies (e.g., wearing textured insoles or compression socks) [14–18]. Therefore, we sought to address the issue of decreased functionality of proprioception, associated with falls in elderly people or those with debilitating conditions such as peripheral neuropathy.

The underlying mechanism behind the enhancement of proprioceptive information from wearing compression garments has been explained by using an ecological dynamics framework. It has been argued that sensorimotor system noise could be enhanced by introducing additional intermittent, intermediate levels of variation in proprioceptive information via wearing compressive and textured materials which compress, contort and deform receptors in the skin surface and joint soft tissue [15, 17, 19, 20]. These materials may counter intuitively help performers to boost information from the background noise to enhance the perception of somatosensory feedback [21]. For example, wearing knee-length clinical level compression socks (> 20 mmHg) has been found to increase the efficiency of sensorimotor integration in older participants when balancing on an unstable surface with eyes open [18]. Cutaneous receptors in the lower limbs convey high fidelity information for joint positioning and play an important role in providing kinesthetic information to regulate behavior during a joint matching task [22, 23]. Wearing non-clinical compression sleeves (< 20 mmHg) was found to increase precision and sensitivity at the joint [24], and improve information on the direction sense of the limbs during movement [25], by filtering the irrelevant mechanoreceptor information and enhancing sensory information related to proprioception [24]. Evidence from these studies implied that sensory feedback was modulated, improving the proprioception regulation of action when wearing clinical and non-clinical compression wearable garments in the lower limbs. Therefore, it is postulated that wearing knee-length compression socks could stimulate the mechanoreceptors and proprioceptors of the lower limb somatosensory system in helping the older adults to make better judgements when positioning their lower limbs in space.

Studies have shown that compression garments improved joint position sense at the ankle and knee joints in young adults [26, 27]. It has been reported that below-knee non-compression, clinical and non-clinical level compression wearable garments benefitted performance of individuals with proprioceptive deficits resulted from injury and age-related degeneration [14, 17, 26, 28, 29]. Similarly, non-compressive garments such as tapes [2, 30, 31], braces and sleeves [32–34] also improved joint position sense at the ankle and knee joints in young adults. These studies found that mean position error was reduced either by wearing a non-compressive or compressive garment during joint position sense tests. An interesting question concerns the plausibility that wearing wearable garments such as socks (compressive or non-compressive) could enhance older people’s proprioception regulation of action in the lower extremities, particularly, below-knee stimulation, with the possibility of reducing trips and falls.

To date, limited empirical studies have investigated the use of compression garments on lower-limb proprioceptive feedback in older adults, with beneficial effects on joint position sense mainly observed in young adults. There has only been one study examining the effects of applying compression bandages in the elderly population. Results showed that application of clinical level compression bandages class II (23–32 mm Hg) at the ankle and foot are associated with improvement of ankle joint position sense [14]. However, Hijman et al. [14] pointed out that the 12 elderly participants were selectively chosen and the results might not be representative for the average population of elderly individuals. Therefore, in this study, we sought to understand whether wearing knee-length compression socks would enhance proprioceptive feedback, providing more accurate information about foot positioning from a larger sample size, in randomly selected older adults. We sought to ascertain the value of wearing compression socks as a possible affordable intervention to minimize the misjudgement movements of foot-ground interactions, especially in individuals with declining sensory and proprioception systems due to ageing. The main purpose of this study was to examine acute effects of wearing knee length socks (KLS) of various compression levels on ankle joint position sense in community-dwelling older adults. It was hypothesized that wearing compression KLS would improve the proprioceptive regulation of foot positioning, specifically ankle joint positioning, in these community-dwelling older adults. We also sought to understand whether there would be any differences in effects on quality of proprioceptive information provided when wearing clinical and non-clinical compression, and non-compression level socks during task performance.

## Methods

### Participants

The procedures used in the study were approved and in accordance with the ethical guidelines of the research ethics committee of Nanyang Technological University, Singapore. Voluntary and informed consent was obtained (written) from all participants. Community-dwelling older adults (12 male and 14 female), aged between 65 and 84 years (mean age: 73.7 ± 5.7 years old; mean height: 1.59 ± 0.08 m; mean weight: 60 ± 10.6 kg; BMI: 23.9 ± 4.5; mean MMSE scores: 26.3 ± 1.9), were randomly recruited from the local senior activity centres in Singapore, who had participated in the profiling of the Singapore community-dwelling older adults project [35]. The detailed breakdown of the participants’ demographic data is shown in Table 1. Specific inclusion criteria are ability to stand independently without using any assistive devices (e.g., walking stick; umbrella), ability to follow instructions, no history of falls, no peripheral neuropathy diseases and brain injuries, Mini-Mental State Examination (MMSE) score > 24 (normal).

**Table 1:** Participants’ demographic.

	Men (N = 12)	Women (N = 14)
Age (years)	76 ± 4.5	71.6 ± 6.0
Height (m)	1.64 ± 0.06	1.55 ± 0.07
Weight (kg)	58.5 ± 11.1	61.2 ± 10.5
BMI	21.9 ± 4.5	25.7 ± 3.9
Mini-Mental State Examination (Scores)	25.6 ± 1.5	26.9 ± 2.1

### Apparatus and tasks

**Fig 1:**
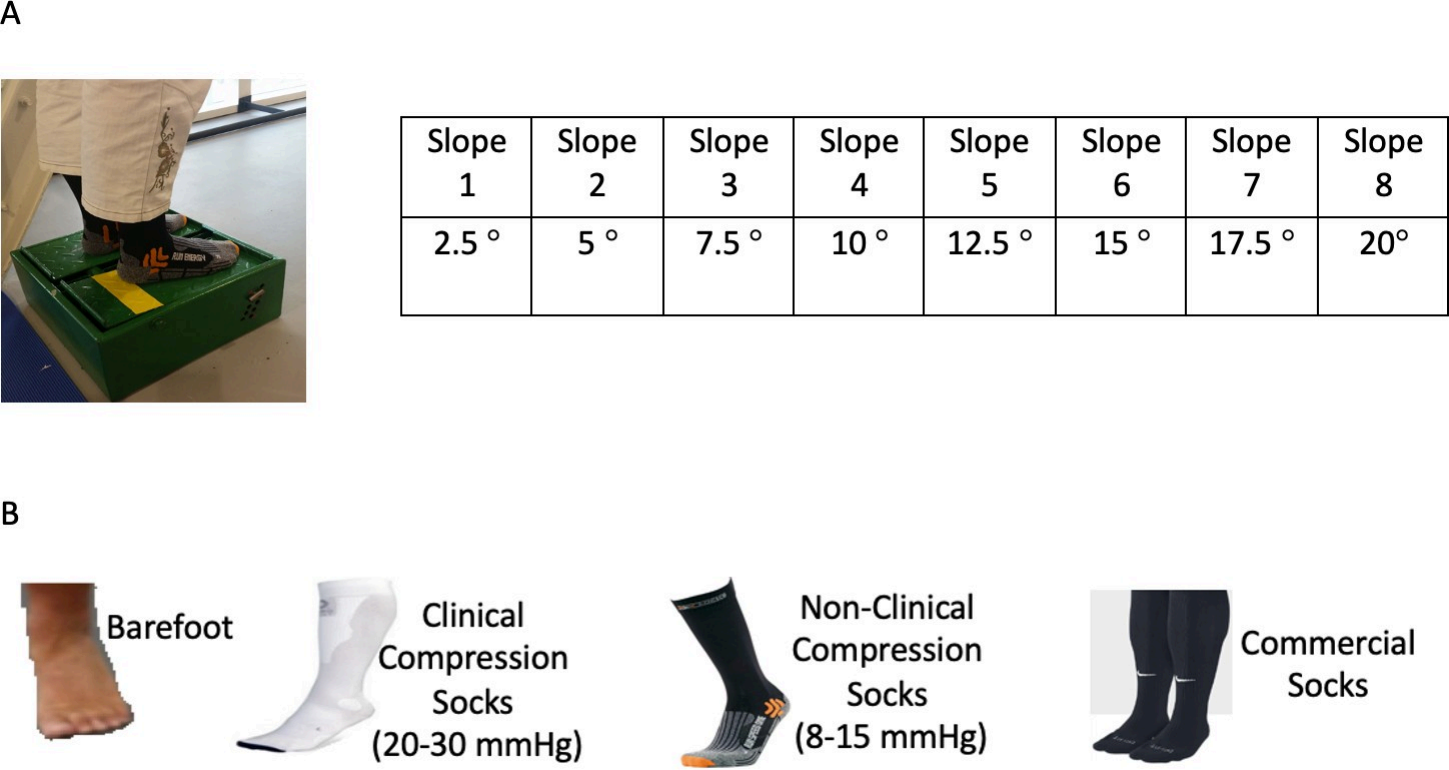
(A). A modified slope box. (B) Barefoot and three different types of socks.

Participants were asked to use the dominant foot to indicate 8 levels of the steepness of incline (2.5°, 5°, 7.5°, 10°, 12.5°, 15°, 17.5°, and 20°) while standing on a customised slope box (Fig 1). The modified slope box of this study was designed based on the direct scaling method developed by Robbins & Waked [36]. The inclination of this box could be altered between 0° and 20° in steps of 2.5° in the direction of plantar flexion. Every level of steepness, in an ascending order, was assigned a number label from 1 to 8. The estimates of surface slopes were based on a ratio scale from 1 to 8, where 1 corresponded to 2.5° and 8 to 20°. This method was purposefully designed to aid the participants to indicate their perception of the slope during actual performance and measurement. This was considered especially important because it has been suggested that plantar flexion position is more prone to functional decline [2].

Three, counterbalanced treatment interventions were provided, while participants wore the following socks: (i) 20–30 mmHg clinical compression (CC) KLS (Zeropoint, Finland); (ii) 8–15 mmHg non-clinical compression (NCC) KLS (X-bionic, Switzerland); (iii) non-compression (NC) models KLS (Mizuno, Japan); with (iv), one control condition (barefoot) (Fig 1). We sought to discern whether the degree of contortion of lower limb tissue, by wearing garments of varying compression levels (compared to a barefoot condition), might influence ankle joint position awareness on different inclines in elderly men and women. All treatment interventions and the 8 levels of incline steepness were presented in a random order for each participant.

### Procedure

Compression socks were assigned to the participants based on the manufacturer’s sizing guidelines. A repeated-measures design was used to determine effects on joint position awareness of four different treatments–wearing CC KLS; wearing NCC KLS; wearing NC KLS; barefoot. The sequence order number for the treatment conditions (Table 2) was assigned randomly to each participant using Microsoft Excel. The levels of steepness were presented in a random, no particular order, during the testing. The randomisation method was used to prevent carryover and order effects.

**Table 2:** The sequence order number for the treatment conditions.

**Sequence order 1:** Bare > CC > NCC > NC	**Sequence order 2:** CC > NCC > NC > Bare	**Sequence order 3:** NC > Bare > CC > NCC	**Sequence order 4:** NCC > CC > NC > Bare
**Sequence order 5:** Bare > NC > CC > NCC	**Sequence order 6:** CC > Bare > NC > NCC	**Sequence order 7:** NC > CC > NCC > Bare	**Sequence order 8:** NCC > NC > Bare > CC

Abbreviation: CC, Clinical compression; NCC, Non-clinical compression; NC, Non-compression; Bare, Barefoot.

During the familiarisation stage, a 1-minute familiarisation period was given to each participant to familiarise themselves with the levels of steepness by using the dominant foot on an adjustable modified slope box before data collection. The dominant foot was determined by asking the participant the leg that they (would) use to kick an object in front of them, like a ball. During the familiarisation period, participants were asked to remember and associate each assigned number that represented each level of steepness. Researchers checked with the participants on perception the steepness of the surface slopes (assigned numbers) while they were sensing and remembering the surface slopes.

During the actual measurement phase, participants were told that an assigned number (surface slope angles) could be repeated, if needed, more than once during each treatment, although that did not happen. Participants were required to stand vertically with zero-degree flexion at the knee, and the specified foot was placed on a designated foot placement area with the toes touching the edge of the box. They were asked to place their body weight on the slope box to feel each position, and to look directly in front and refrain from any eye contact with the modified slope box. Researchers would then randomly adjust the slope box, in no particular order, to various levels of steepness until all levels of steepness had been presented. After every adjustment, participants were asked to indicate the perceived level of steepness, by indicating the assigned number, when researchers presented them with the randomly selected target position. The same process would then be repeated for the second trial. Participants were asked to hold on to the railing beside them for safety purposes between trials. Participants were given a sufficient rest period (~ 2 minutes) between each treatment.

### Data processing and statistical analysis

The absolute error value was positive whether the error was recorded as an under- or over-estimation of the target position. This measure indicated the precision of estimates [36]. The constant error value refers to the direction of position errors from the target position (i.e., a positive value for overestimation and negative for underestimation). Constant error was used to measure the direction of imprecision. For each treatment and the barefoot condition, the average errors of both trials were used for statistical analysis of the data. SPSS software (version 26.0) was used for statistical analysis. All data were analyzed by using parametric statistical tests as the data were normally distributed. Cronbach’s Alpha coefficient was used to check the reliability of the joint position sense test for the three treatment conditions, with the barefoot condition serving as the baseline, across the surface slopes tested. A value of 0.7 to 0.8 is an acceptable value for Cronbach’s Alpha [37]. A repeated measure Analysis of Variance (ANOVA) with two within-participant factors on treatments and levels of surface slopes was administered. Analyses of the main effects of treatment and levels of surface slope conditions were corrected using a Bonferroni adjustment. Alpha level was set at <0.05 for all statistical analyses.

## Results

### Reliability test

For both absolute and constant errors measurement, the Cronbach’s alpha value showed good reliability of the joint position test for the three treatment conditions compared with the control condition (i.e., barefoot) (Table 3).

**Table 3:** Cronbach’s Alpha.

	Cronbach’s Alpha (average measures)		
	CC-Bare	NCC-Bare	NC-Bare
Constant errors	0.74	0.83	0.76
Absolute errors	0.74	0.74	0.82

Abbreviation: CC, Clinical compression; NCC, Non-clinical compression; NC, Non-compression; Bare, Barefoot.

### Absolute errors

**Fig 2:**
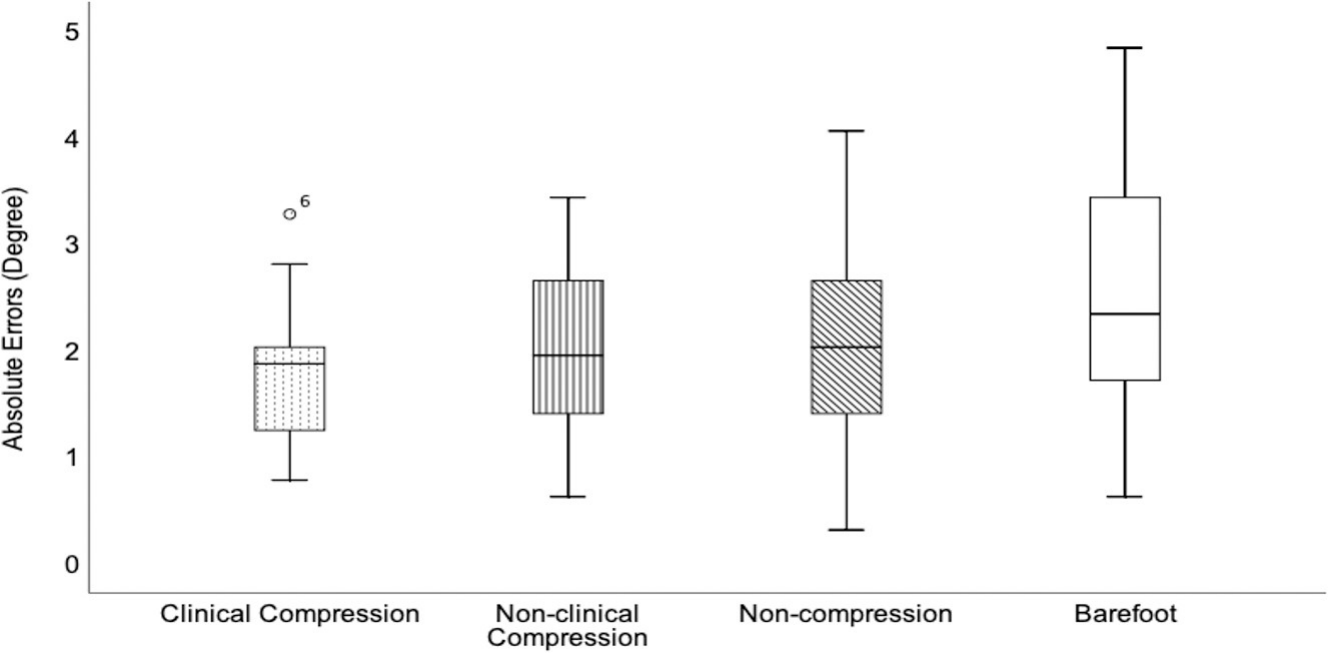
Boxplot data representative of the four conditions.

Table 4 shows the mean absolute errors of the three treatments condition. For mean absolute errors, there was a significant main effect of treatment intervention F (3, 75) = 4.086, p = 0.01, η_p_^2^ = 0.14). Pairwise comparison revealed that wearing clinical compression socks significantly reduced the mean absolute errors compared to the barefoot condition (p = 0.031). However, there were no significant differences between clinical KLS and other KLS (non-clinical KLS; normal KLS), and between other KLS and barefoot conditions (p > 0.05). The boxplot shows the range of scores, the range between the middle 50% of scores fall, the median, the upper and lower quartile scores of the three treatments and barefoot condition (Fig 2). The range of absolute errors scores of clinical compression socks was smaller than other treatment conditions and the barefoot condition. This shows that more consistent effects were seen in the participants who wore clinical compression socks. The median score for clinical compression is lower than treatment intervention and barefoot condition, which informed us that wearing clinical compression is more effective in reducing the perception errors.

**Table 4:** Mean and standard deviation (SD) values for absolute errors during the four treatment conditions.

	Absolute Errors			
Conditions	Mean	SD	95% Confidence Interval	
			Lower Bound	Upper Bound
Clinical Compression Socks	1.8*	0.67	1.53	2.07
Non-clinical Compression Socks	2.04	0.78	1.73	2.36
Normal Socks	1.98	0.89	1.63	2.34
Barefoot	2.47	1.06	2.04	2.9

* Significantly lower than barefoot.

### Constant errors

**Fig 3:**
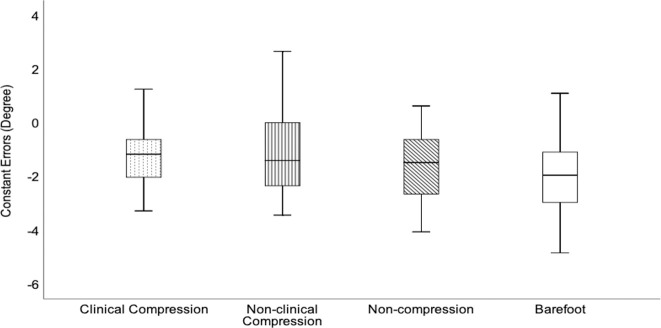
Boxplot data representative of the four conditions.

Table 5 shows the mean constant errors of the three treatments and barefoot condition. There was no significant main effect of treatments intervention (F (3, 75) = 2.032, p > 0.05, η_p_^2^ = 0.075). The boxplot data show that the range of constant errors was the smallest for clinical compression socks (Fig 3). The median score was lower than other treatment intervention and barefoot condition. Overall, the results revealed a trend of wearing socks in helping the older adults to reduce the possibility of underestimating the slope surface.

**Table 5:** Mean and standard deviation (SD) values for constant errors during the four treatment conditions.

	Constant Errors			
Conditions	Mean	SD	95% Confidence Interval	
			Lower Bound	Upper Bound
Clinical Compression Socks	-1.22	1.03	-1.63	-0.81
Non-clinical Compression Socks	-1.21	1.53	-1.83	-0.59
Normal Socks	-1.50	1.22	-2.00	-1.01
Barefoot	-1.92	1.48	-2.52	-1.32

## Discussion

The purpose of this study was to examine acute effects of wearing knee-length socks (KLS) of various compression levels on ankle joint position awareness in community-dwelling healthy older adults. Compared to other treatment interventions and barefoot condition, our results showed that wearing clinical compression knee-length socks reduced mean absolute error significantly in the ankle joint (plantar flexion) awareness test. This suggests that wearing clinical compression KLS could reduce the estimation errors in the ankle joint. Second, we observed that the median error score was lower, and a smaller range of variability was observed in participants who were wearing clinical compression KLS. Third, the findings showed positive (but not statistically significant) trend of wearing compression KLS for the direction of estimation of ankle joint with a reduction in the degree of underestimation. With a significant finding of using clinical compression KLS and the positive trend shown in wearing KLS, the findings might be taken to imply the possibility that wearing KLS could have boosted perception of somatosensory information from the lower limbs in older adults.

We found that wearing KLS improved the precision of estimation of ankle joint plantar flexion movement, especially the clinical compression KLS, by reducing the absolute errors in a representative sample of older adults. Consistent with findings reported by Hijman et al. (2009), this study supports the idea that the use of clinical compression socks could reduce the ankle joint perceptual errors in elderly participants wearing compression garments [14]. In agreement with our findings, Ghai et al. (2016) found that wearing below-knee compression garments improved joint proprioception, by reducing the mean errors of a repositioning task [26]. In addition, our study showed small variability in the spread of the joint position sense error data in participants wearing the clinical compression KLS. This functional role of variability induced in the sensorimotor system by wearing clinical compression socks could act as a form of “essential noise” in neurobiological systems which can help individuals to regulate their actions when negotiating the environment [16]. The results of the current study support the possibility that wearing KLS, especially clinical compression socks, could improve joint sensitivity and precision and enhance somatosensory feedback that emerges from pressure on cutaneous and joint receptors in the lower legs [7, 14, 17, 20, 23, 24].

However, the evidence on effects of wearing KLS on the direction of positioning errors in the joint position sense (JPS) task was inconclusive. Most of our findings showed that wearing KLS reduced constant errors, but this observation did not lead to comparisons that crossed the conventional threshold of statistical significance. The underestimation of surface slope observed in the elderly sample could be an indication of the inadequacy of muscle contraction, with ageing, in providing information to support the feet for a required foot position [7]. Doan et al. (2003) found that compression garments reduced muscle oscillation which optimizes the neurotransmission and mechanics at the molecular level of muscle contraction [38]. We postulate that wearing KLS could have stimulated the muscle spindles and mechanoreceptors along the shank. This could possibly enhance the functionality of muscle contraction, thereby reducing the level of constant limb positioning errors as observed in our sample of older adults. While the current findings are interesting, more studies are needed to confirm the potential effects of KLS especially the compression socks on the functionality of muscle contraction.

On the other hand, we observed that participants produced relatively larger absolute and constant errors in the barefoot condition compare to KLS condition. A wider range of absolute errors was also observed when participants performed the task in a barefoot condition. This observation might suggest that without lower-limb stimulation support, such as using KLS, the degeneration of the somatosensory system may have led to some inconsistencies in providing accurate perception during the joint positioning test. This finding might be based on neural activity from lower limbs becoming less efficient due to the changes in cutaneous sensitivity and receptor morphology in ageing somatosensory systems. More studies with a larger sample size are needed to further explore the efficiency of wearing KLS, particularly with compression, in guiding the ankle towards more precise positioning.

A potential limitation of this study includes the absence of an assessment of the overall comfort of wearing knee-length socks and it is unknown whether individual comfortability affected performance outcome, if at all. It was found that overall comfortability of wearing wearable garments to be positively associated with actual proprioceptive performance [39]. A second limitation could be the levels of the working memory and attentional load required on this memory-based joint position matching tasks. There was the possibility of the older adults guessing the stages which might have affected the results, although our occasional checks did not imply that participants were guessing. To avoid such issue in future studies, researchers could reduce the number of stages of the slopes and use visual representation of the angles instead of using the assigned corresponding number. The last limitation of this study could be the use of a modified slope box with passive JPS test instead of a Biodex System Isokinetic Dynamometer with an active JPS test. The modified slope box was chosen due to perceived low education levels of the participants and thus a possible lack of understanding of the concept of angles by the participants. For future studies, researcher could conduct a similar study with a group of more educated older adults who would be more familiar with the concept of angles. In addition, more studies are needed to investigate effects of wearing KLS on the direction of imprecision in limb positioning in the elderly.

Nevertheless, we want to highlight the strengths of the study. First, minimal logistics were involved and the testing protocol was practical and simple for older adults to follow. This process reduces the anxiety and uneasiness in older adults which they often feel uncomfortable when asked to interact with a mechanical device. Second, the use of a randomisation approach was helpful in sequencing the order of intervention treatments (e.g., Clinical compression KLS, Non-clinical compression KLS, Non-compression KLS, Barefoot) and the order of presentation of ankle joint movements (e.g., 2.5°, 5.0°, 7.5°, 10.0°, 12.5°, 15.0°, 17.5°, 20.0°). The randomization of the sequence orders prevented carry-over effects for participants. Therefore, this method minimizes the risk of bias in research investigation and improves confidence in the quality of the outcome data.

In summary, proprioception regulation of action in our sample participants was improved by the use of clinical compression KLS for the plantar flexion position. The findings of this study suggested that wearing knee-length socks could have boosted perception of somatosensory information from the lower limbs, amplifying the information to regulate action from muscle and cutaneous receptors, thereby improving proprioception in older adults. Wearing knee-length socks could potentially form an affordable strategy to ameliorate negative effects of ageing on the proprioception system in older adults. Further research is needed to refine understanding of the potential benefits of wearable garments on proprioception in older individuals. Future studies could also examine the long-term effects of wearing knee length socks on various ranges of ankle joint positions (e.g., dorsi flexion and inversion).

## Supporting information

S1 Table Absolute and constant errors data. (XLSX)

## References

[1] Gilman S. Joint position sense and vibration sense: anatomical organisation and assessment. Journal of Neurology, Neurosurgery, and Psychiatry. 2002;73(5):473–7. 10.1136/jnnp.73.5.473 12397137 PMC1738112

[2] Iris M, Monterde S, Salvador M, Salvat I, Fernández-Ballart J, Judith B. Ankle taping can improve proprioception in healthy volunteers. Foot & Ankle International. 2010;31(12):1099–106. 10.3113/FAI.2010.1099 21189212

[3] You SH. Joint position sense in elderly fallers: A preliminary investigation of the validity and reliability of the SENSERite measure. Archives of Physical Medicine and Rehabilitation. 2005;86(2):346–52. 10.1016/j.apmr.2004.01.035 15706567

[4] Suetterlin KJ, Sayer AA. Proprioception: where are we now? A commentary on clinical assessment, changes across the life course, functional implications and future interventions. Age and Ageing. 2014;43(3):313–8. 10.1093/ageing/aft174 24231586

[5] Waddington G, Adams R. Football boot insoles and sensitivity to extent of ankle inversion movement. British Journal of Sports Medicine. 2003;37(2):170 10.1136/bjsm.37.2.170 12663362 PMC1724612

[6] Ko S, Simonsick E, Deshpande N, Ferrucci L. Sex-specific age associations of ankle proprioception test performance in older adults: results from the Baltimore Longitudinal Study of Aging. Age and Ageing. 2015;44(3):485–90. 10.1093/ageing/afv005 25637144 PMC4411223

[7] Robbins S, Waked E, McClaran J. Proprioception and stability: foot position awareness as a function of age and footwear. Age and Ageing. 1995;24(1):67 10.1093/ageing/24.1.67 7762465

[8] Thelen DG, Brockmiller C, Ashton-Miller JA, Schultz AB, Alexander NB. Thresholds for Sensing Foot Dorsi- and Plantarflexion During Upright Stance: Effects of Age and Velocity. The Journals of Gerontology. Series A, Biological Sciences and Medical Sciences. 1998;53(1):M33–8. 10.1093/gerona/53A.1.M33 9467431

[9] Goble DJ, Coxon JP, Wenderoth N, Van Impe A, Swinnen SP. Proprioceptive sensibility in the elderly: Degeneration, functional consequences and plastic-adaptive processes. Neuroscience and Biobehavioral Reviews. 2009;33(3):271–8. 10.1016/j.neubiorev.2008.08.012 18793668

[10] Siong K, Kwan MM, Lord SR, Lam AK, Tsang WW, Cheong AM. Fall risk in Chinese community-dwelling older adults: A physiological profile assessment study. Geriatrics & Gerontology International. 2016;16(2):259–65. 10.1111/ggi.12463 25655079

[11] Shaffer SW, Harrison AL. Aging of the somatosensory system: a translational perspective. Physical Therapy. 2007;87(2):193–207. 10.2522/ptj.20060083 17244695

[12] Lord SR, Fitzpatrick RC. Choice stepping reaction time: a composite measure of falls risk in older people. The Journals of Gerontology. Series A, Biological Sciences and Medical Sciences. 2001;56(10):M627–32. 10.1093/gerona/56.10.M627 11584035

[13] Ribeiro F, Oliveira J. Aging effects on joint proprioception: the role of physical activity in proprioception preservation. European Review of Aging and Physical Activity. 2007;4(2):71–6. 10.1007/s11556-007-0026-x

[14] Hijmans JM, Zijlstra W, Geertzen JHB, Hof AL, Postema K. Foot and ankle compression improves joint position sense but not bipedal stance in older people. Gait & Posture. 2009;29(2):322–5. 10.1016/j.gaitpost.2008.10.051 19019679

[15] Orth D, Davids K, Wheat J, Seifert L, Liukkonen J, Jaakkola T, et al The role of textured material in supporting perceptual-motor functions. PLoS One. 2013;8(4):e60349 10.1371/journal.pone.0060349 23565232 PMC3615024

[16] Qiu F, Cole MH, Davids KW, Hennig EM, Silburn PA, Netscher H, et al Enhanced somatosensory information decreases postural sway in older people. Gait & Posture. 2012;35(4):630–5. 10.1016/j.gaitpost.2011.12.013 22245163

[17] Woo MT, Davids K, Liukkonen J, Orth D, Chow JY, Jaakkola T. Effects of different lower-limb sensory stimulation strategies on postural regulation—A systematic review and meta-analysis. PLoS One. 2017;12(3):e0174522 10.1371/journal.pone.0174522 28355265 PMC5371369

[18] Woo MT, Davids K, Liukkonen J, Chow JY, Jaakkola T. Immediate effects of wearing knee length socks differing in compression level on postural regulation in community-dwelling, healthy, elderly men and women. Gait & Posture. 2018;66:63–9. 10.1016/j.gaitpost.2018.08.011 30165286

[19] Hasan H, Davids K, Chow JY, Kerr G. Compression and texture in socks enhance football kicking performance. Human Movement Science. 2016;48:102–11. 10.1016/j.humov.2016.04.008 27155962

[20] Woo MT, Davids K, Liukkonen J, Jaakkola T, Chow JY. Effects of textured compression socks on postural control in physically active elderly individuals. Procedia Engineering. 2014;72:162–7. 10.1016/j.proeng.2014.06.028

[21] Davids K, Shuttleworth R, Button C, Renshaw I, Glazier P. "Essential noise"—enhancing variability of informational constraints benefits movement control: a comment on waddington and adams. British Journal of Sports Medicine. 2003;38:601–5. 10.1136/bjsm.2003.007427 15388548 PMC1724948

[22] Edin BB. Cutaneous afferents provide information about knee joint movements in humans. The Journal of Physiology. 2001;531(1):289–97. 10.1111/j.1469-7793.2001.0289j.x 11179411 PMC2278439

[23] Lowrey CR, Strzalkowski NDJ, Bent LR. Skin sensory information from the dorsum of the foot and ankle is necessary for kinesthesia at the ankle joint. Neuroscience Letters. 2010;485(1):6–10. 10.1016/j.neulet.2010.08.033 20817078

[24] Barss TS, Pearcey GE, Munro B, Bishop JL, Zehr EP. Effects of a compression garment on sensory feedback transmission in the human upper limb. Journal of Neurophysiology. 2018;120(1):186–95. 10.1152/jn.00581.2017 29641310 PMC6093963

[25] Born D, Sperlich B, Holmberg H. Bringing Light into the Dark: Effects of Compression Clothing on Performance and Recovery. International Journal of Sports Physiology and Performance. 2013;8(1):4–18. 10.1123/ijspp.8.1.4 23302134

[26] Ghai S, Driller MW, Masters RSW. The influence of below-knee compression garments on knee-joint proprioception. Gait & Posture. 2016;60:258–61. 10.1016/j.gaitpost.2016.08.008 27523397

[27] You SH, Granata KP, Bunker LK. Effects of circumferential ankle pressure on ankle proprioception, stiffness, and postural stability: a preliminary investigation. The Journal of Orthopaedic and Sports Physical Therapy. 2004;34(8):449 10.2519/jospt.2004.34.8.449 15373008

[28] Birmingham TB, Kramer JF, Kirkley A, Inglis JT, Spaulding SJ, Vandervoort AA. Knee bracing after ACL reconstruction: effects on postural control and proprioception. Medicine and Science in Sports and Exercise. 2001;33(8):1253–8. 10.1097/00005768-200108000-00002 11474323

[29] Ghai S, Driller M, Masters RSW. Effects of joint stabilizers on proprioception and stability: A systematic review and meta-analysis. Physical Therapy in Sport. 2016;25:65–75. 10.1016/j.ptsp.2016.05.006 28262354

[30] Simoneau GG, Degner RM, Kramper CA, Kittleson KH. Changes in ankle joint proprioception resulting from strips of athletic tape applied over the skin. Journal of Athletic Training. 1997;32(2):141–7. 16558444 PMC1319817

[31] Robbins S, Waked E, Rappel R. Ankle taping improves proprioception before and after exercise in young men. British Journal of Sports Medicine. 1995;29(4):242–7. 10.1136/bjsm.29.4.242 8808537 PMC1332234

[32] Herrington L, Simmonds C, Hatcher J. The effect of a neoprene sleeve on knee joint position sense. Research in Sports Medicine. 2005;13(1):37–46. 10.1080/15438620590922077 16389885

[33] Van Tiggelen D, Coorevits P, Witvrouw E. The effects of a neoprene knee sleeve on subjects with a poor versus good joint position sense subjected to an isokinetic fatigue protocol. Clinical Journal of Sport Medicine. 2008;18(3):259–65. 10.1097/JSM.0b013e31816d78c1 18469568

[34] Wu KH, Ng YF, Mak FT. Effects of knee bracing on the sensorimotor function of subjects with anterior cruciate ligament reconstruction. The American Journal of Sports Medicine. 2001;29(5):641–5. 10.1177/03635465010290051801 11573924

[35] Woo MT, Davids K, Liukkonen J, Chow JY, Jaakkola T. Falls, cognitive function, and balance profiles of Singapore community-dwelling elderly individuals: key risk factors. Geriatric Orthopaedic Surgery & Rehabilitation. 2017;8(4):256–62. 10.1177/2151458517745989 29318089 PMC5755848

[36] Robbins S, Waked E. Foot position awareness: The effect of footwear on instability, excessive impact, and ankle spraining. Critical Reviews of Physical and Rehabilitation Medicine. 1997;9(1):53–74.

[37] Field A. Discovering statistic using SPSS (3rd Ed.)—and sex and drugs and rock ’n’ roll. London: SAGE; 2009.

[38] Doan B, Kwon YH, Newton R, Shim J, Popper E, Rogers R, et al Evaluation of a lower-body compression garment. Journal of Sports Sciences. 2003;21(8):601–10. 10.1080/0264041031000101971 12875311

[39] Long Z, Wang R, Han J, Waddington G, Adams R, Anson J. Optimizing ankle performance when taped: Effects of kinesiology and athletic taping on proprioception in full weight-bearing stance. Journal of Science and Medicine in Sport. 2016;20(3):236–40. 10.1016/j.jsams.2016.08.024 27686616

